# Immune Mechanisms Responsible for Vaccination against and Clearance of Mucosal and Lymphatic Norovirus Infection

**DOI:** 10.1371/journal.ppat.1000236

**Published:** 2008-12-12

**Authors:** Karen A. Chachu, Anna D. LoBue, David W. Strong, Ralph S. Baric, Herbert W. Virgin

**Affiliations:** 1 Department of Pathology and Immunology, Washington University School of Medicine, St. Louis, Missouri, United States of America; 2 Department of Microbiology and Immunology, School of Medicine, University of North Carolina at Chapel Hill, Chapel Hill, North Carolina, United States of America; 3 Department of Molecular Microbiology, Washington University School of Medicine, St. Louis, Missouri, United States of America; Baylor College of Medicine, United States of America

## Abstract

Two cardinal manifestations of viral immunity are efficient clearance of acute infection and the capacity to vaccinate against secondary viral exposure. For noroviruses, the contributions of T cells to viral clearance and vaccination have not been elucidated. We report here that both CD4 and CD8 T cells are required for efficient clearance of primary murine norovirus (MNV) infection from the intestine and intestinal lymph nodes. Further, long-lasting protective immunity was generated by oral live virus vaccination. Systemic vaccination with the MNV capsid protein also effectively protected against mucosal challenge, while vaccination with the capsid protein of the distantly related human Lordsdale virus provided partial protection. Fully effective vaccination required a broad immune response including CD4 T cells, CD8 T cells, and B cells, but the importance of specific immune cell types varied between the intestine and intestinal lymph nodes. Perforin, but not interferon gamma, was required for clearance of MNV infection by adoptively transferred T lymphocytes from vaccinated hosts. These studies prove the feasibility of both mucosal and systemic vaccination against mucosal norovirus infection, demonstrate tissue specificity of norovirus immune cells, and indicate that efficient vaccination strategies should induce potent CD4 and CD8 T cell responses.

## Author Summary

Human noroviruses are the most common cause of epidemic nonbacterial gastroenteritis in the world. Despite their importance as human pathogens, little is known about how the immune system controls and clears norovirus infection, and the potential and mechanisms of vaccination remain unclear. Here, we used norovirus infection of mice to show that vaccination can provide long-lasting immunity against mucosal norovirus challenge and to identify the types of immune cells that are important in vaccination against norovirus infection. Similarly, we identified the types of immune T cells that are important for clearance of acute infection. Efficient vaccination required all three major arms of adaptive immunity: CD4 T cells, CD8 T cell, and B cells. Importantly, protective vaccination against mucosal challenge was observed after either mucosal or systemic norovirus antigen exposure. The pore-forming molecule perforin was important for T cell-mediated control of norovirus infection. Our study has important implications for understanding adaptive immunity to norovirus infection, and may provide insight into the directions to take in developing a human norovirus vaccine.

## Introduction

More than 90% of epidemic nonbacterial gastroenteritis worldwide can be attributed to human noroviruses (HuNV) [1]–[3]. Infection is transmitted fecal-orally, and symptomatic infection is characterized by nausea, vomiting and/or diarrhea lasting 24–48 hours within 24 hours of exposure [4]. Despite the significant costs and morbidity of HuNV infections, no vaccine is currently available. The elderly and individuals in long-term care facilities may be more susceptible to either norovirus infection or norovirus-induced disease [5] and would be an important target population for a norovirus vaccine. The reasons for increased incidence and/or susceptibility to HuNV disease are unknown. This is due in part to our incomplete understanding of norovirus immunity. The potential to vaccinate against these and related viruses has been demonstrated in gnotobiotic piglets, cats and rabbits [6]–[8], but the immune mechanisms responsible have not been identified. The challenges for vaccine efficacy may be very different between different caliciviruses. For example, variation in MNV strains is significantly less than between HuNV strains [9]. Human volunteer studies demonstrate short-term, but not long-term, protection against homologous, but not heterologous, viral challenge [10]–[12]. Since HuNV belong to 3 genogroups (GI, GII and GIV) with many strains in each genogroup [4], this lack of cross-protection is a challenge for vaccine development. Frequent exposure to noroviruses within short time periods stimulates sustained immunity and resistance to norovirus induced illness [13],[14]. Serum antibody levels in adults reflect susceptibility to infection and do not always correlate with protection [13],[14]. In children, however, serum antibody levels correlate with protection, likely reflecting short-term immunity and recent exposure [15]–[17]. A nonfunctional fucosyl transferase gene (FUT2) accounts for a significant proportion, though not all, of resistance to Norwalk virus infection, suggesting that other factors, yet undiscovered, may contribute to norovirus resistance [18],[19].

In the absence of a cell culture system for HuNV, virus like particles (VLPs) that assemble when the viral capsid protein is expressed have been important for evaluating norovirus immune responses [20]–[23]. Studies using Norwalk Virus (GI), Snow Mountain Virus (GII) and HuNoV-HS66 (GII) VLPs to evaluate immunity after infection with live virus or immunization with VLPs orally show production of T cell effector cytokines such as IL-2 and interferon γ (IFN-γ) and proliferation of norovirus specific T cells after *in vitro* restimulation with VLPs [24]–[26]. These studies show that T cell responses develop, but do not define their role in either clearance of primary infection or resistance to re-challenge. Together, they suggest the potential for vaccination, but leave open important questions about the effectiveness and longevity of vaccine immune responses, mechanisms of vaccination, the viral protein targets for protective responses, and the potential for cross-protection between distantly related noroviruses.

The identification of the first murine norovirus, MNV, and its propagation in cultured cells provides a facile animal model for studies of norovirus immunity and pathogenesis [27],[28]. MNV, an enteric virus that infects tissues of the gastrointestinal tract, is spread by the fecal-oral route ([27] and unpublished studies). The MNV genome encodes four open reading frames. ORF1 encodes a polyprotein that is cleaved into individual non-structural proteins similar to the polyprotein of HuNV [29]. ORF2 encodes the major capsid protein VP1 and ORF3 encodes a minor capsid protein. The existence of a protein product for ORF4 has not been confirmed. In the MNV virion structure, the capsid, like that of human noroviruses, consists of 90 dimers of VP1 [30]. There are differences between the MNV virion and previously reported VLP structures. The MNV protruding domain is lifted off the shell domain by approximately 16 Angstroms and rotated approximately 40 degrees in a clockwise fashion, forming interactions at the P1 base in an infectious virion that have not been observed previously. The existence of these novel aspects of the structure are consistent with the hypothesis that MNV may undergo a capsid maturation process [30].

Studies of MNV pathogenesis reveal an important role for interferon (IFN) and STAT-1 mediated innate immunity in resistance to infection and MNV induced lethality [27],[31]. The importance of adaptive immunity in control of MNV infection is indicated by the observation that RAG1-/- mice develop persistent MNV infection while wild type (WT) mice can clear infection with some strains of MNV [9],[27],[31].

While MNV is an efficient enteric virus that infects many mice in research mouse colonies around the world, diarrhea has not been reported after MNV infection. Thus, MNV provides an infection only model for HuNV infection. Viral titers in tissues of infected mice have not been reported to exceed 10^6^ PFU/ml, and this highest level of viral titer is obtained after infection of highly susceptible STAT1-/- mice [31]. In RAG1-/- mice and WT mice, viral titers of 10^2^ to 10^4^ PFU/ml are routinely observed [9],[31]. The availability of a plaque assay for MNV allows the analysis of MNV infection despite these low titers. Some MNV strains persist at a low level in WT mice, while others are cleared from intestine, spleen, liver, mesenteric lymph nodes (MLN) and feces within 7 days of infection [9],[27],[31]. Additionally, in wild type C57BL6/J mice MNV replicates maximally in the distal ileum [9], in comparison to wild type 129S6/SvEvTac mice where replication occurs in the proximal intestine [31]. The significance of these differences is not known.

Studies of norovirus infection in human volunteers have not specifically investigated whether the infection spreads beyond the intestine to the local lymph nodes, however, it is possible that systemic invasion occurs in humans with chronic conditions or immunosuppressed hosts [32]–[36]. Additionally, viremia has been reported in infections of gnotobiotic pigs and calves [26],[37],[38]. Thus, the ability of MNV to spread to tissues other than the intestine after oral infection may not be unique, but the relationship of this aspect of MNV pathogenesis to human infection is not clear. The availability of strains that can be cleared from WT mice, such as MNV1.CW3, provides an opportunity to define the mechanisms responsible for two cardinal aspects of viral immunity: the capacity to effectively clear acute infection and the immune mechanisms responsible for effective vaccination.

B cells and MNV specific antibody are important in the clearance of primary MNV infection [39], but the role of T cells in clearance and the potential and mechanisms of vaccination against mucosal norovirus challenge are unknown. We show here that vaccination with either live MNV or Venezuelan Equine Encephalitis replicon particles (VRPs) expressing the MNV capsid protein VP1 protect the intestine against re-challenge for at least six months. Live virus was more effective than VRP-mediated vaccination. There was partial cross protection against MNV infection after vaccination with a HuNV capsid protein. We found that both the clearance of primary infection and vaccination require the concerted efforts of CD4 T cells, CD8 T cells, B cells, and that T cells required the effector molecule perforin for maximal impact on MNV infection. The effects of specific immune cell types were tissue specific, differing between ileum and mesenteric lymph nodes. These are the first studies to demonstrate immune mechanisms responsible for norovirus clearance and vaccination.

## Results

### Short-Term Live Virus and Subunit Vaccination against MNV

We first determined whether we could detect short-term immunity to homologous MNV challenge and whether proteins encoded by specific MNV ORFs could elicit effective immunity. VRPs expressing ORF1, ORF2 and ORF3 of MNV1.CW3 and ORF2 of the HuNV Lordsdale (genogroup GII.4) and Chiba (genogroup GI.4) were produced for vaccination experiments. Western blots of VRP-infected cell lysates revealed proteins of appropriate sizes [29],[40] and additionally showed that hyper-immune polyclonal rabbit antisera to MNV [28] cross-reacted at low levels with VLPs from Chiba virus and Lordsdale virus (Figure S1A).

**Figure 1:**
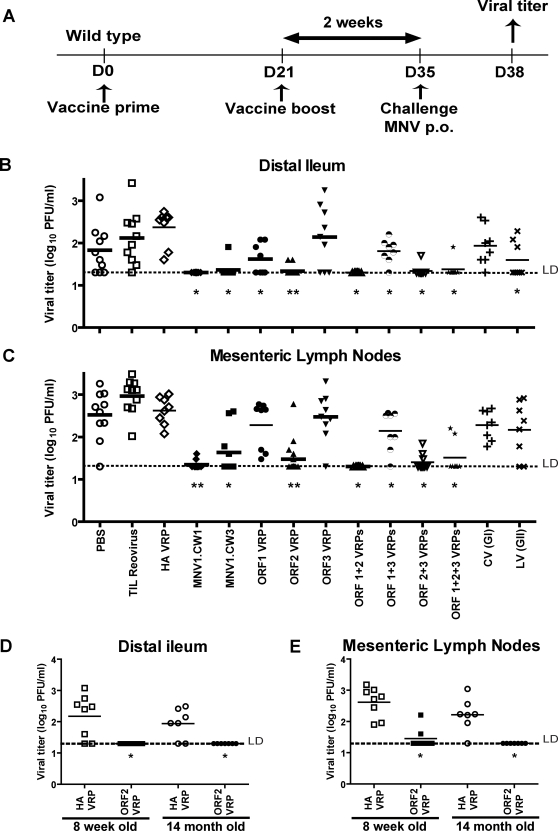
Short-term vaccination against MNV using live MNV strains and VRPs expressing ORF1, ORF2, and ORF3 from MNV and ORF2 from Chiba virus and Lordsdale virus. (A) Vaccination protocol used in short-term vaccination. Viral titers in (B) distal ileum and (C) MLN of adult (8-week-old) WT mice after MNV1.CW3 challenge following vaccination with the indicated vaccines. Viral titers in (D) distal ileum and (E) MLN of adult (8-week-old) or aged (14-month-old) WT mice immunized with MNV1.CW3 ORF2 VRP or HA VRP and challenged with MNV1.CW3. These data are pooled from two independent experiments with 3-5 mice per group in each experiment. (**) indicates p<0.0001, (*) indicates p<0.05. Unless otherwise stated, live virus vaccinations are compared to vaccination with reovirus, and VRP vaccinations are compared to HA VRP. LD indicates the limit of detection. Bars indicate the arithmetic mean.

WT mice were vaccinated and boosted three weeks later. Two weeks after boosting, mice were challenged with MNV1.CW3 and organs titered for MNV three days later (Figure 1A). In these WT mice, maximal MNV replication in the intestinal tract occurs in the distal ileum [9] and viral titers could not be detected in duodenum/jejunum (data not shown). After oral inoculation with MNV1.CW3, WT mice exhibit detectable viral titers in the distal ileum and the MLN three to five days post-infection [9],[31]. Prior infection with either MNV1.CW1 (p = 0.0002) or MNV1.CW3 (p = 0.0009) significantly decreased MNV1.CW3 replication in the distal ileum compared to control mice infected with reovirus (Figure 1B). Similar decreases were observed in the MLN after vaccination with MNV1.CW1 (p = 0.0001) or MNV1.CW3 (p = 0.0003) (Figure 1C) compared to the reovirus controls. Similar results were observed in the spleen (data not shown). There was no statistically significant difference between vaccination with MNV1.CW1 or MNV1.CW3. This demonstrates that a protective secondary immune response develops after clearance of primary MNV infection.

ORF2 VRPs protected against MNV1.CW3 in both distal ileum (p = 0.005) and MLN (p = 0.02) compared to control VRPs expressing hemagglutinin (HA) from a mouse adapted influenza A virus [41] (HA VRP control group). Controls for VRP vaccination also included PBS. HA VRP controls were not significantly different from PBS controls across all experiments and statistical comparisons for VRP vaccination are therefore shown to HA VRP controls. ORF1 VRPs alone in the distal ileum, or in both the distal ileum and MLN when combined with ORF3 VRPs, had a small but statistically significant effect on MNV1.CW3 levels (Figure 1B and 1C). ORF3 VRPs alone did not confer significant protection (Figure 1B and 1C). Together these data show that vaccination with either live virus or ORF2 VRPs can confer short-term protection against MNV challenge.

We next assessed vaccination with heterologous ORF2 proteins. Mice were vaccinated and boosted with VRPs expressing ORF2 from Chiba Virus or Lordsdale virus and challenged with MNV1.CW3. Vaccination with Lordsdale virus capsid led to statistically significant protection against MNV infection in the distal ileum, (p = 0.0007, Figure 1B) but not the MLN (Figure 1C). No significant reduction in MNV titers was seen after immunization with Chiba virus capsid (Figure 1B and 1C). Protection after Lordsdale ORF2 VRP vaccination did not correlate with generation of cross-reactive serum IgG in these mice, measured by ELISA, despite the potential for such cross-reactivity revealed by western blot (Figure S1B). Fecal extracts from immunized mice yielded no measurable homotypic or heterotypic IgG or IgA (data not shown). Taken together, these data show that there is measurable functional immunologic cross protection between Lordsdale virus and MNV in the distal ileum. The lack of a correlation between serum or fecal antibody responses and protection suggested that protection may be T cell mediated.

### Vaccination Can Occur in Aged Mice

Since older adults may be more susceptible than younger adults to norovirus infection or disease [5], we determined whether increased age altered vaccine efficacy. Prior work has shown that mice older than 1 year of age have diminished vaccine responses to SARS virus antigens [42]. We therefore compared vaccine efficacy in adult (8 week old) and aged (14 month old) mice. Adult and aged mice were vaccinated and challenged as before. In contrast to studies using SARS virus antigens [42], aged mice responded as well as adult mice to MNV ORF2 vaccination in both the distal ileum and MLN (Figure 1D and 1E). Despite this protective effect, sera from vaccinated aged mice had significantly lower anti-MNV ORF2 IgG compared to adult mice (Figure S1C). These data indicated that protection against MNV infection occurred in the absence of robust serologic responses, again raising the possibility that T cells play a fundamentally important role in vaccination against MNV.

### Protective Effect of Live MNV Vaccine Was Sustained over Six Months

**Figure 2:**
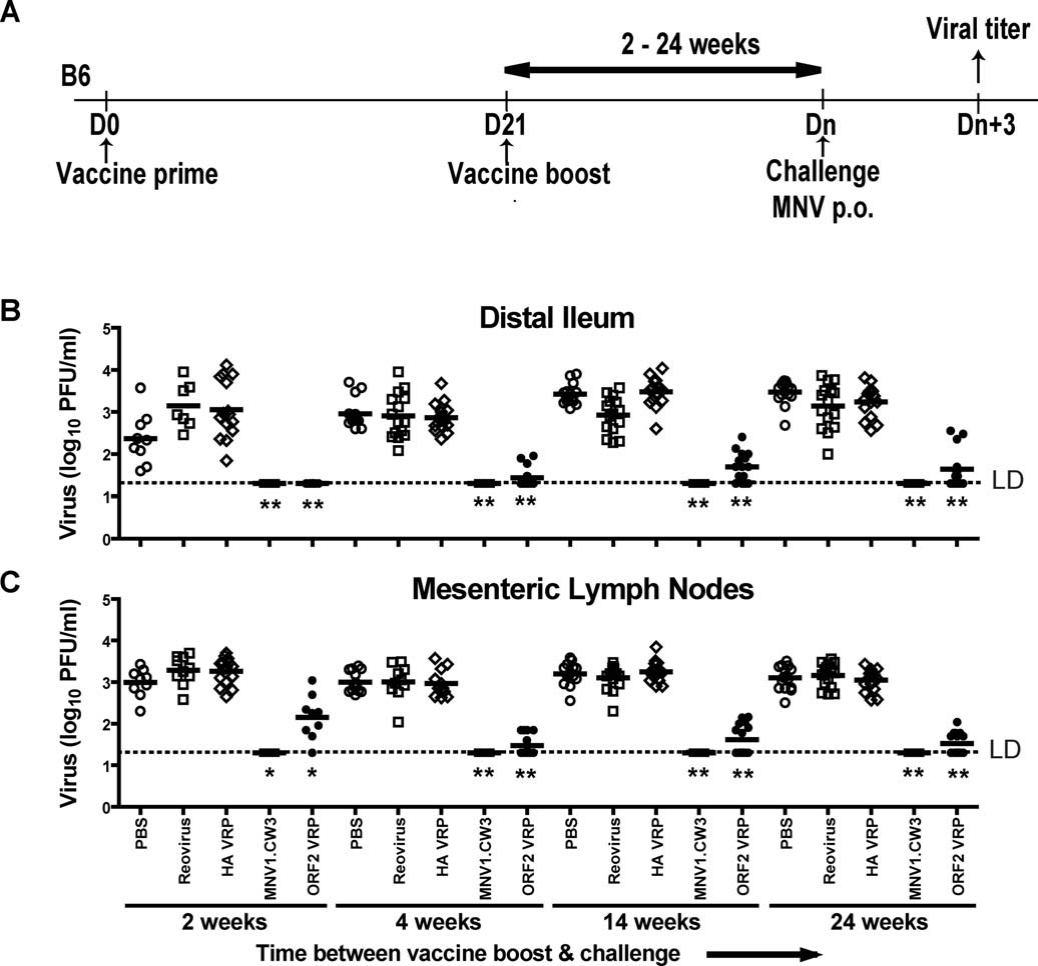
Long-term vaccination against MNV using a live MNV strain and ORF2 VRPs. (A) Vaccination protocol used in long-term vaccination. Viral titers in (B) distal ileum and (C) MLN of WT mice after MNV1.CW3 challenge following vaccination with indicated vaccines. These data are pooled from three independent experiments with 3–5 mice per group in each experiment. (**) indicates p<0.0001, (*) indicates p<0.05. Unless otherwise stated, live virus vaccinations are compared to vaccination with reovirus, and VRP vaccinations are compared to HA VRP. LD indicates the limit of detection.

We next determined whether protection conferred by MNV1.CW3 or MNV ORF2 VRPs was long lived. WT mice were primed and boosted as shown in Figure 2A with MNV1.CW3 or MNV ORF2 VRPs. Mice were then challenged with MNV1.CW3 two, four, 14, or 24 weeks later and MNV titers measured three days post-challenge. Two weeks post-boost, we observed complete protection against ileal MNV1.CW3 infection after vaccination with either MNV1.CW3 (p = 0.0001) or ORF2 VRPs (p<0.0001) compared to reovirus or HA VRP controls (Figure 2B). At two weeks, while vaccination with either MNV1.CW3 or ORF2 VRPs limited MNV1.CW3 replication in MLN, live virus vaccination was more effective (p<0.0001) (Figure 2C). Live virus vaccination conferred full protection against MNV1.CW3 replication in both the distal ileum and the MLN at four, 14 and 24 weeks after vaccine boost. Vaccination with ORF2 VRPs was also protective, albeit less effective than vaccination with MNV1.CW3 (Figure 2B and 2C). Thus both live virus and subunit vaccine induce long-term protection against MNV infection, with live virus vaccination providing more complete protection.

### Mechanisms Responsible for Vaccination by Live Virus and ORF2 VRPs

**Figure 3:**
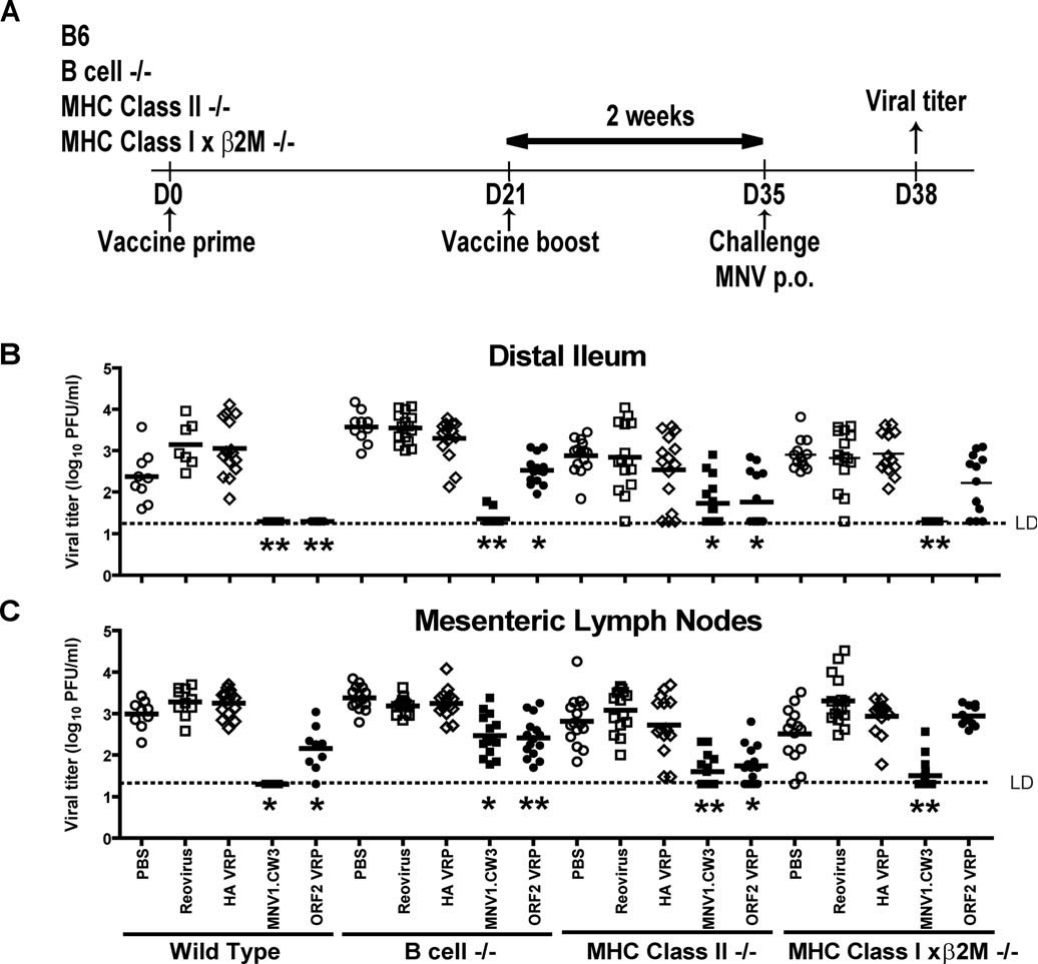
Complete short-term protection against MNV infection requires MHC Class II, MHC Class I, β2M, and B cells. (A) Vaccination protocol used in short-term vaccination using immunodeficient mice. Viral titers in (B) distal ileum, (C) MLN of B cell-/, MHC Class II-/-, and MHC Class I×β2M-/- mice after MNV1.CW3 challenge following short-term vaccination with the indicated vaccines. These data are pooled from three independent experiments with 3–5 mice per group in each experiment. (**) indicates p<0.0001, (*) indicates p<0.05. Unless otherwise stated, live virus vaccinations are compared to vaccination with reovirus, and VRP vaccinations are compared to HA VRP. LD indicates the limit of detection.

We next determined the mechanism(s) responsible for effective vaccination. We vaccinated mice lacking both major histocompatibility complex (MHC) Class I and β2 microglobulin (β2M) [43] (CD8 T cells deficiency [44]), MHC Class II (CD4 T cells deficiency [45]), or B cell deficient mice [46] (Figure 3A). These experiments were conducted concurrently with the experiments in Figure 2 above, as such the data from WT mice are repeated in the figure for comparison. Live MNV vaccination induced significant protection against MNV challenge in both the distal ileum and the MLN of B cell-/-, MHC Class II-/- and MHC Class I×β2M-/- mice (p<0.05 in all cases, Figure 3B and 3C). However, there was considerable variation in the efficacy of vaccination in distal ileum and MLN between different immunodeficient strains. In B cell-/- mice, after vaccination with live virus, only 2 out of 15 mice had any titer (and those two mice had less than 100 PFU of MNV) and in MHC Class I×β2M-/- mice, similar vaccination led to undetectable viral titers in the distal ileum (Figure 3B) but detectable titers in the MLN (Figure 3C). In MHC Class II-/- mice, there were detectable titers in both tissues (Figure 3B and 3C). Results for ORF2 vaccination showed that protection required the activity of all major aspects of the adaptive immune response (Figure 3B and 3C). Moreover, there was no protection elicited by ORF2 vaccination in either intestine or MLN tissue after vaccination of MHC Class I×β2M-/- mice with ORF2 VRPs (Figure 3B and 3C) indicating that protection by VRPs critically depends on CD8 T cells. These data demonstrated that complete protection in all tissues after vaccination with live virus required the concerted actions of B cells, MHC Class II, MHC Class I and β2M. Further, the results were consistent with tissue specific roles for B cells, CD4 T cells and CD8 T cells in the development of complete protection against MNV infection.

### CD8 and CD4 T Cells Are Important for Clearance of Primary MNV Infection

**Figure 4:**
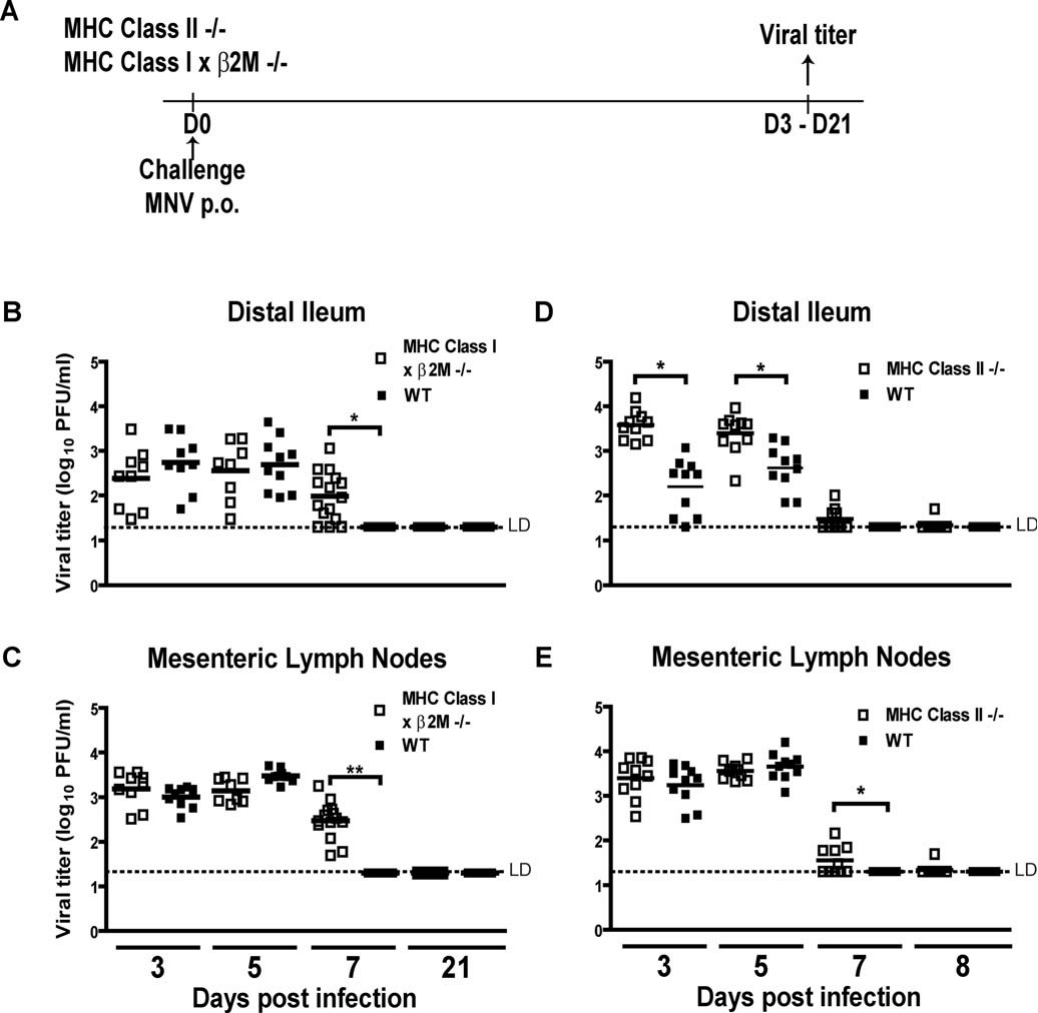
MHC Class II limits early MNV replication, and deficiency in MHC Class II or MHC Class I & β2M delays MNV clearance. (A) Protocol of challenge used in experiments in this figure. Viral titers in distal ileum (B,D) and MLN (C,E) of WT and MHC Class I×β2M-/- mice (B,C) and MHC Class II-/- mice (D,E) after infection with MNV1.CW3. These data are pooled from two to three independent experiments with 3–5 mice per group in each experiment. (**) indicates p<0.0001, (*) indicates p<0.05. LD indicates the limit of detection.

We next determined whether the same cell types that were required for vaccination were also required for efficient clearance of acute infection. We focused on the role of T cells in clearance since the role of B cells in clearance has already been demonstrated [39]. To determine the role of T cells in clearance of acute MNV infection we inoculated WT, MHC Class II-/-, and MHC Class I×β2M-/- mice orally with MNV1.CW3 and measured viral titers in the distal ileum and MLN three, five, seven and 21 days post-infection (Figure 4B–4E).

There was no significant difference in viral titer between MHC Class I×β2M-/-mice and WT mice at three and five days post-infection, indicating that MHC Class I and β2M were not required in MNV infection at early time points (Figure 4B and 4C). However, at seven days post-infection, MHC Class I×β2M-/- mice had significant levels of MNV titers in both the distal ileum (p = 0.0002) and the MLN (p<0.0001) compared to WT mice, which had completely cleared the infection (Figure 4B and 4C). MHC Class I×β2M-/- mice eventually cleared MNV infection, demonstrated by the lack of viral titers at 21 days post-infection. Thus, MHC Class I and β2M, and by inference CD8 T cells were important for efficient clearance of MNV, but were not required for eventual clearance of MNV infection.

In contrast to MHC I×β2M-/- mice, MHC Class II-/- mice had higher MNV titers in the ileum than WT mice both three (p = 0.0002) and five (p = 0.0058) days after infection (Figure 4D). At seven days post-infection, minimal viral titers remained and by eight days post-infection, both MHC Class II-/- and WT mice had cleared the infection from the distal ileum. In MLN, viral titers in WT and MHC Class II-/- were not significantly different at days three and five post-infection. However, there was a small, but statistically significant increase in titer in the MLN of MHC Class II-/- compared to WT mice at seven days post-infection (p = 0.04, Figure 4E). By eight days post-infection, MLN infection was cleared. Together these data indicated that MHC Class II, and by inference CD4 T cells, were necessary for control of acute MNV infection but are not required for eventual clearance of MNV infection.

**Figure 5:**
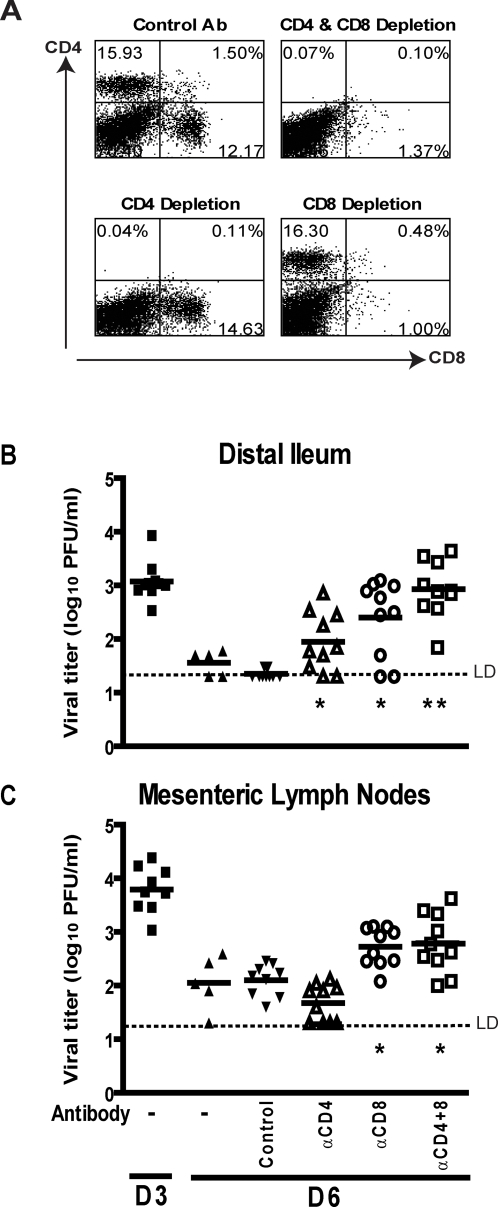
CD4 and CD8 T cells are required for clearance of primary MNV infection at 6 days post-infection. (A) Representative flow cytometric analysis of CD4 and CD8 staining on splenocytes harvested from control and immunodepleted WT mice 6 days post-infection with MNV1.CW3. Viral titer in (B) distal ileum and (C) MLN after treatment with the indicated antibodies. Results are pooled from two independent experiments with 3–5 mice per group in each experiment. (**) indicates p<0.0001, (*) indicates p<0.05. LD indicates the limit of detection.

To exclude the possibility that the phenotypes we observed in MHC Class I×β2M-/- and MHC Class II-/- mice were due to abnormal immune ontogeny in knockout mice, we determined the requirement for CD4 and CD8 T cells in the clearance of primary MNV infection in WT mice depleted of CD4 and CD8 T cells. Depletion of CD4 and CD8 T cells was at least 90% effective as assessed by flow cytometry of isolated splenocytes (Figure 5A) and this depletion protocol is effective at depleting T cells in secondary lymphoid organs and the intestine [47],[48]. In comparison to control antibody, depletion of CD4 T cells, led to a significant increase in MNV titers in the distal ileum (p = 0.0053, Figure 5B), but not the MLN (Figure 5C). In contrast, depletion of CD8 T cells led to an increase in MNV titers in both the distal ileum (p = 0.004, Figure 5B), and the MLN (p = 0.0025, Figure 5C).

Together, these data from primary challenges of non-immune mice lacking antigen presenting molecules or depleted of specific T cell subsets demonstrated that CD4 T cells are important for efficient MNV clearance in the distal ileum especially at days three and five, while their role in the MLN is small. CD8 T cells are important for efficient clearance of MNV infection in both the MLN and distal ileum, and they function later in infection than CD4 T cells, being most important at days six and seven.

### Function of CD4 and CD8 T Cells from Immunized Mice

**Figure 6:**
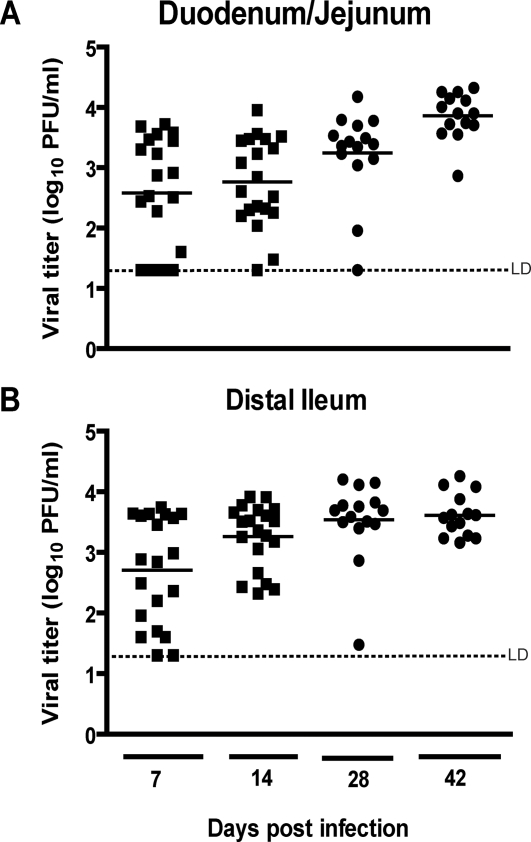
RAG1-/- mice fail to clear MNV infection in the intestine. Viral titers in (A) duodenum/jejunum and (B) distal ileum of RAG1*-/-* mice after infection with MNV1.CW3. Results are pooled from four independent experiments with 5 mice per group in each experiment. LD indicates the limit of detection.

We next determined whether CD4 and CD8 T cells from vaccinated mice can, alone or in combination, clear MNV infection from mucosal sites. We have previously shown that MNV infected RAG1-/- mice have high levels of viral RNA present in multiple tissues up to 90 days post-infection [27]. We therefore determined MNV viral titers in RAG1-/- mice. By 42 days post-infection, all RAG1-/- mice had consistent, high levels of MNV in both duodenum/jejunum and distal ileum (Figure 6A and 6B), as well as several other tissues (data not shown). These data confirmed that mice lacking adaptive immunity fail to clear MNV infection [27].

**Figure 7:**
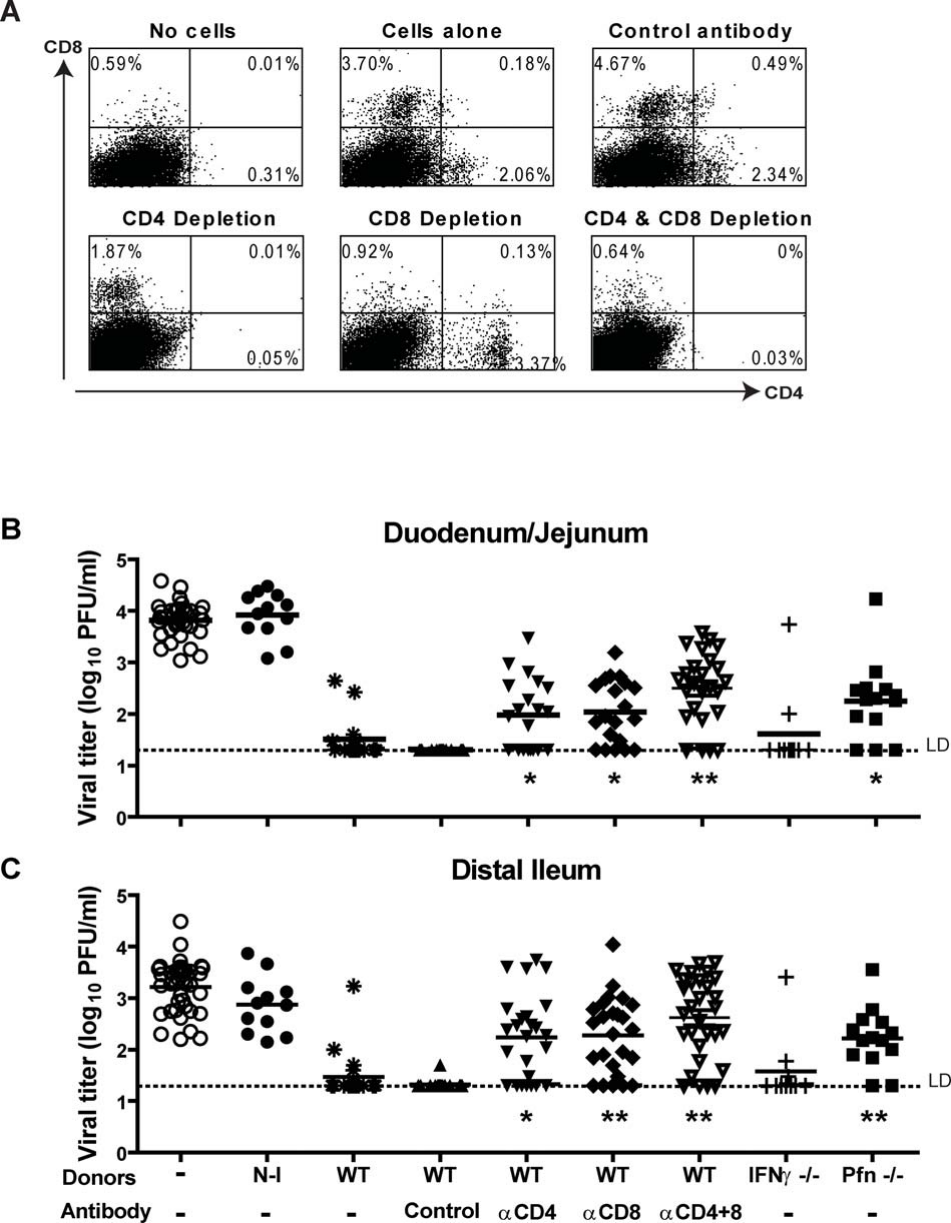
Immune CD4 and CD8 T cells are both required, and perforin plays a role in clearance of persistent MNV infection in RAG1-/- recipients. (A) Representative flow cytometric analysis of splenocytes harvested from RAG1-/- recipient mice 6 days post-transfer of splenocytes. Viral titers in (B) duodenum/jejunum and (C) distal ileum 6 days after adoptive transfer of medium alone, WT non-immune splenocytes (N–I), WT immune splenocytes (WT), WT immune splenocytes with or without depleting antibodies or immune splenocytes from IFNγ-/- or perforin-/- (Pfn-/-) mice. These data are pooled from three independent experiments with 3–5 mice per group in each experiment. (**) indicates p<0.0001, (*) indicates p<0.05. LD indicates the limit of detection.

The availability of persistently infected RAG1-/- mice allowed us to determine the role of CD4 and CD8 T cells in clearance of MNV infection using adoptive transfer of splenocytes from MNV immune WT mice into persistently infected RAG1-/- mice. Transfer of immune, but not non-immune, splenocytes significantly reduced MNV titer in the duodenum/jejunum (p<0.0001) and distal ileum (p<0.0001) six days post-transfer (Figure 7B and 7C). Thus, adoptively transferred immune splenocytes were sufficient to clear persistent MNV infection in the intestine of RAG1-/- mice.

To define which cells were required for MNV clearance, CD4 or CD8 T cells were depleted from splenocytes transferred into RAG1-/- recipients. Anti-T cell antibodies effectively depleted the appropriate T cell populations, as measured six days post-transfer by flow cytometry (Figure 7A). Depletion of either CD4 or CD8 T cells individually led to a significant increase in MNV titers in duodenum/jejunum compared to control depletion (Figure 7B, CD4 depletion p = 0.0042; CD8 depletion p = 0.0002). Depletion of both CD4 and CD8 T cells from transferred immune splenocytes caused a significant additional increase in MNV titers when compared to either CD4 depletion alone (p = 0.02) or CD8 depletion alone (p = 0.03). In the distal ileum, depletion of either CD4 T cells (p = 0.0003) or CD8 T cells (p<0.0001) led to a significant increase in MNV titers (Figure 7C). These data demonstrated that both immune CD4 and CD8 T cells were necessary for clearance of persistent MNV infection from the intestine.

### Perforin Has a Role in Clearance of MNV Infection

Two major effector mechanisms for the antiviral effects of T cells are the production of IFNγ and perforin mediated cytolysis [49]. We therefore adoptively transferred immune splenocytes from IFNγ-/- or perforin-/- mice into persistently infected RAG1-/- mice and determined their capacity to clear intestinal MNV infection. Immune splenocytes from IFNγ-/- mice were as effective as those from WT mice (Figure 7B and 7C). However, immune splenocytes from perforin-/- mice were less effective at clearing MNV infection from the duodenum/jejunum (p = 0.0003, Figure 7C) or distal ileum (p = 0.0075, Figure 7B) than cells from either WT or IFNγ-/- mice, but more effective compared to transfer of non-immune cells in duodenum/jejunum (p = 0.0086) or distal ileum (p = 0.0001). Thus, while perforin was critical for efficient clearance of MNV infection from the intestine, it was not the only relevant effector mechanism.

## Discussion

In this paper we define the mechanisms of immunity to norovirus infection as measured by both vaccination against, and clearance of, mucosal infection. We found that it is possible to generate highly effective, and remarkably long lasting, immunity to norovirus infection by oral exposure to live virus. Further, systemic exposure to the viral capsid protein expressed in a vaccine vector resulted in effective immunity, albeit not as effective as that observed after live virus vaccination. Importantly, this shows that the MNV VP1 protein contains relevant B cell, CD4 T cell and CD8 T cell epitopes. Vaccination was effective in aged mice. Additionally, vaccination in adult mice required the concerted action of CD4 T cells, CD8 T cell, and B cells to be completely protective in the tissues surveyed. Interestingly, the activities of different components of the adaptive immune system in clearance and vaccination were tissue specific, with different cells playing roles in the intestine itself compared to the draining lymph nodes. Perforin was an important effector molecule. These data have important implications for understanding adaptive immunity to an animal norovirus, representative of a genus that causes significant disease in humans.

HuNV infection and disease is rapid, with symptoms developing within 24–48 hours of infection and lasting for a few days. Thus, we selected three days after challenge as a readout for infection in our studies, since relevant vaccine-generated immune responses would have to act very early after challenge. Lack of any of the three components of the adaptive response: B cells, CD4 T cells, or CD8 T cells significantly diminished vaccine effects generated by either live virus or VP1 capsid protein immunization, and delayed viral clearance during primary infection. This indicates that VP1 has antibody epitopes as well as MHC H-2b restricted CD4 and CD8 T cell epitopes. These data suggest that it may be necessary to engage the concerted actions of an intact immune response including both MHC Class I and MHC Class II restricted T cells and antibody responses to efficiently vaccinate against HuNV infection.

The protection against MNV infection in aged mice in the absence of robust generation of anti-MNV antibodies raised the possibility that an important component of the vaccine response is T cell dependent, a hypothesis borne out in adoptive transfer studies. Importantly, the antiviral effector perforin is important in the clearance of MNV from the intestine, suggesting that the cytotoxic T cell response is a key effector mechanism. It is possible that other cell types such as NK cells might also use perforin as a mechanism to control MNV infection. Our data do not rule out a role for IFNγ in clearance of MNV infection since NK cells in recipient RAG1-/- mice can make IFNγ, but do suggest that T cell derived IFNγ plays at most a minor role in effector T cell function in the ileum. This argues that classical CTL assays may be a good surrogate for the development of effective vaccine-generated immune responses to HuNV.

Live virus vaccination was more effective than VRP based vaccination. The lower level of protection that we observed with ORF2 VRPs in contrast to MNV1.CW3 may be due to many factors, and this study does not provide mechanistic insights into this difference. In comparison to VLPs, VRPs may have advantages in systemic vaccination including targeting dendritic cells and intrinsic adjuvant activities [50]. These properties of VRPs may be responsible for the effectiveness of systemic single protein subunit vaccination against mucosal viral challenge in this case. However, it may be that because VRPs undergo a single round of replication at the site of inoculation they cannot generate the same breadth of immunity that is generated by live replicating virus. While VRP vaccination clearly induces some relevant effector and memory cell responses, vaccination with capsid alone may not sufficient to generate the complete antigenic repertoire required for effective immunity. Interestingly, we found some protection with the non-structural ORF1 polyprotein, suggesting that protective epitopes exist outside of the capsid protein. As the ORF1 polyprotein is expressed early after infection, it may be that these epitopes would be valuable targets for generating an efficient immune response.

Of note, vaccination with VP1 via the subcutaneous route provided significant protection despite the fact that the vaccination occurred systemically, while protection was read out at a mucosal site. This indicates that an active systemic immune response can provide protection against norovirus infection, and a mucosal vaccine may not be necessary to vaccinate against norovirus infection. Importantly, systemic vaccination was dependent on T cells, indicating that the relevant cells can traffic to the intestine after peripheral VRP-based vaccination.

These studies leave several important questions unanswered. Firstly, we used a homologous virus challenge. In nature, it is likely that hosts are repeatedly challenged with antigenically distinct noroviruses. However, the mouse norovirus strains identified so far fall into a single genogroup, GV, which likely represents a single serotype [9]. In this way murine noroviruses identified to date may present less of a challenge for the immune system than HuNV, which are distributed across 3 genogroups and appear to evolve under antibody selection [51]. In addition, we selected a strain of MNV that is cleared by WT mice. Other strains persist for prolonged periods of up to 35 days [9]. It remains to be determined whether vaccination will be effective against persistent MNV strains. It is interesting that human noroviruses can persist beyond the time frame of usual clinical symptoms [52]–[55]. Long-term persistence might contribute to explaining the sporadic epidemics of infection in the absence of an animal reservoir. Antigenic and pathogenetic complexity will likely be a major issue for the development of norovirus vaccines. The lack of comparable variation in MNV strains limits the utility of the MNV model for assessing immunity to antigenically distinct strains. Perhaps this limitation will be overcome as additional strains of MNV are identified, sequenced, and studied. However, the fact that we observed partial cross protection between MNV and one HuNV, and the demonstration that vaccination with many different VLPs can enhance generation of cross reactive antibodies [56] provide some encouragement.

There are two ways in which murine norovirus infection may not represent the same biology as HuNV infection. The first is the lack of diarrhea in mice infected with the strains of MNV used here. It is possible that the adaptive responses that clear MNV from the intestine demonstrated here are irrelevant to the responses that may prevent human disease. In this regard, it is important to note that studies of adult mice with rotaviruses (also an infection only model), have been important to our considerations of rotavirus vaccines [57]. Importantly, human studies may not reveal the mechanisms of effective immunity and are based on surrogate assays of immunity, since invasive sampling of tissues may be technically difficult. Studies in piglets may be revealing since piglets develop diarrhea when infected with the HuNV strain, HuNoV-HS66 [37]. However, it is more difficult to study immune mechanisms in this system. Thus, we are left with several imperfect systems for considering what one should seek in a HuNV vaccine. Our studies in mice argue for a vaccine that induces all aspects of the adaptive immune response, and that assays for cytotoxic lymphocyte responses to HuNV infection may be an important surrogate assay for protection.

The second aspect of murine norovirus infection that is of unknown relevance to human infection is the impressive capacity of MNV to infect lymph nodes draining the intestine (this paper and [31],[58],[59]). This may be related to the tropism of MNV for dendritic cells and macrophages [28],[59] and likely reflects spread of MNV directly from the intestine, but may also reflect seeding of the MLN from systemic sites. Considering the distal ileum alone, B cells and MHC Class I and β2M were not required for live virus vaccination, and there was significant, but incomplete, protection in MHC Class II-/- mice (Figure 3B and 3C). Consistent with this, studies of primary clearance showed that any single arm of the adaptive response was dispensable for ultimate control of primary infection in the intestine. However, vaccination-mediated control of infection in the MLN, and clearance of primary infection from the MLN [39], required B cells. This differential requirement for components of the immune response in different organs raises an important question about norovirus pathogenesis and lymphoid infection: are the cells infected in intestine and MLN the same? Differences in viral tropism in the two tissues might explain the differential requirement for B cells between ileum and MLN, indicating the importance of future studies on the role of immunity in norovirus cell and organ tropism.

## Materials and Methods

### Viruses, Viral Stocks, VRPs, Plaque Assays

MNV strains MNV1.CW3 or MNV1.CW1 were used in all virus infections [9],[28],[31]. Two mutations (that result in changes in the encoded amino acids) distinguish the genomes of MNV1.CW3 and MNV1.CW1 [28]. To generate a concentrated virus stock, RAW 264.7 cells (ATCC, Manassas, VA) were infected in VP-SFM media (Gibco, Carlsbad, CA) for 2 days at a multiplicity of infection (MOI) of 0.05. Supernatants were clarified by low-speed centrifugation for 20 min at 3,000 rpm. Virus was concentrated by centrifugation at 4°C for 3 h at 27,000 rpm (90,000 *g*) in a SW32 rotor. Viral pellets were resuspended in PBS and titered on RAW 264.7 cells as previously described [28]. Type I Lang reovirus was kindly provided by Dr. Terrence S. Dermody (Vanderbilt University, Nashville, TN). Plaque assays were performed as previously described [28] with the following modifications. Tissues were harvested into sterile, screw-top 2-ml tubes containing 500 µl of 1-mm zirconia/silica beads (BioSpec Products, Bartlesville, OK) and stored at −80°C. To obtain viral titers in these tissues 1 ml of complete DMEM was added to each sample on ice and homogenized using a MagNA Lyser (Roche Applied Science, Hague Road, IN) prior to plaque assay. The limit of detection was 20 plaque forming units (PFU)/ml.

All VRPs were produced as previously described [60]. Briefly, ORFs 1, 2 and 3 from MNV1.CW3 and ORF2 from Lordsdale virus and Chiba virus were each cloned into VRP expression vectors. Following infection of BHK cells with VRPs for 24 h, culture supernatants were harvested and cells lysed. Proteins were separated by SDS-PAGE and analyzed by western blot with polyclonal rabbit anti-MNV serum [28]. VRP titers and efficient expression of recombinant protein were determined by immunofluorescence assay using mouse antisera generated from inoculation with respective antigens. Cell lysates from MNV ORF2, Chiba virus and Lordsdale virus VRP-infected cell cultures were further purified to obtain VLPs [56].

### Cell Cultures and Antibodies

RAW 264.7 cells were maintained as previously described [28]. Monoclonal antibodies (MAbs) specific to CD4 (YTS191.1 [61]), CD8 (H35 [62]) and SFR3-DR5 (ATCC HB-151 [63]) were produced from hybridoma cell lines in INTEGRA Celline CL1000 flasks (Integra Biosciences, Ijamsville, MD) using CD Hybridoma media (Gibco, Carlsbad, CA) as previously described [64].

### Mice, Inoculations, and Infections

All mice were bred and housed at Washington University School of Medicine or the University of North Carolina at Chapel Hill in accordance with all federal and university policies. Wild type C57BL6/J (hereafter referred to as WT, Jackson # 000664), B6RAG1-/- (RAG1-/-, Jackson # 002216), IFNγ-/- (IFNγ-/- , Jackson # 002287), perforin-/- (perforin-/-, Jackson # 002407), MHC Class II-/- (MHC Class II-/-, Jackson #003584) and B-cell-deficient mice backcrossed onto a C57BL/6 background (B cell-/-, Jackson # 002288) mice were purchased from Jackson Laboratory (Bar Harbor, ME). MHC Class II deficient mice (MHC Class II-/-, Taconic #ABBN12-M) and their WT controls C57BL/6Ntac (WT, B6 Taconic) were purchased from Taconic (Germantown, NY). K^b−/−^×D^b−/−^×β2M-/- [43] (MHC Class I×β2M-/-) mice were a generous gift of Dr. Ted Hansen (Washington University, St Louis, MO). For some studies, WT C57BL6/J mice were purchased from Harlan Sprague Dawley (Indianapolis, IN) and aged to 14 months. All mice (or cage sentinel mice for mice deficient in antibody production) were tested by ELISA for the presence of MNV antibody prior to experiments [27]. All mice used in these studies were seronegative at the initiation of experiments.

Mice used in vaccination studies were immunized with 3×10^7^ PFU of MNV1.CW1 [28], MNV1.CW3 [31], or control Type I Lang reovirus per orally (p.o.) in 25 µl of DMEM containing 10% fetal bovine serum (Hyclone, Logan, UT) (cDMEM). VRP immunizations were with 2.5×10^6^ infectious units (IU) of each VRP expressing MNV1.CW3 ORF1, ORF2, or ORF3 individually or in groups of 2–3 VRPs; Chiba virus ORF2 or Lordsdale virus ORF2 in 10 µl or 50 µl volume by footpad inoculation (into the subcutaneous space) [65] on day 0 and boosted on day 21. HA VRP and PBS immunizations in 10 µl or 50 µl volume by footpad inoculation [65] on day 0 and boosted on day 21 served as controls for all VRP immunizations. Mice were challenged with 3×10^7^ PFU of MNV1.CW3 at specified times after boost and tissues harvested three days post-challenge. Controls for VRP vaccination included PBS or VRPs expressing hemagglutinin (HA) from a mouse adapted influenza A virus [41]. PBS served as a control for HA VRP in these experiments in the event that HA VRP had a significant effect on MNV replication. HA VRP controls were not significantly different from PBS controls in all experiments and both are presented in all figures for completeness.

RAG1-/- and all splenocyte donor mice were infected with 3×10^6^ PFU of MNV1.CW3 p.o. in 25 µl of cDMEM. All other mice were infected with 3×10^7^ PFU MNV1.CW3 p.o. In RAG1-/- mice two segments of the small intestine were harvested: a one inch section of the small intestine immediately distal to the pylorus of the stomach, (designated the duodenum/jejunum), and a one inch section of the small intestine immediately proximal to the cecum (designated the distal ileum). In all other mice the distal ileum and three mesenteric lymph nodes (MLN) were harvested. With the exception of RAG1-/- mice (inoculated at 4–6 weeks of age) and aged WT mice (inoculated at 14 months of age), all other mice were inoculated at 6–10 weeks of age.

### Adoptive Transfer Studies

Spleens were harvested from mice and single cell suspensions were generated [65]. Cells were counted and diluted in RPMI-1640 media (Sigma, Saint Louis, MO) supplemented with 10% fetal calf serum (HyClone, Logan, UT), 100 U penicillin/ml, 100 µg/ml streptomycin, 10 mM HEPES (N-2-hydroxyethylpiperazine-N9-2-ethanesulfonic acid), 1mM sodium pyruvate, 50 µM 2-mercaptoethanol and 2 mM L-glutamine (cRPMI). In all adoptive transfer experiments, 1×10^7^ cells were injected into persistently infected RAG1-/- mice by intraperitoneal (i.p.) injection in 0.5ml cRPMI.

### In Vivo Depletion of Lymphocyte Subsets

For depletions in WT mice, 500 µg of lymphocyte-depleting antibody or an isotype-matched control antibody [SFR3-DR5, IgG2b] was administered i.p. one day prior and one day after infection. For depletions in adoptive transfer experiments, depleting antibodies were administered to RAG1-/- recipients as described above with one dose one day prior to splenocyte transfer and a second dose on the day of trnsfer. The efficacy of lymphocyte depletion in both sets of depletion experiments was monitored by flow cytometric analysis of splenocytes at the end of the experiment.

### MNV ELISA

ELISA to detect binding of polyclonal anti-serum or fecal extract-derived antibody to purified MNV virions or MNV VLPs was performed as previously described [27],[56].

### Statistical Methods

All data were analyzed using GraphPad Prism software (GraphPad Software, San Diego, CA). Viral titer data were analyzed with the nonparametric Mann-Whitney test. All differences not specifically stated to be significant were insignificant (*p*>0.05).

## Supporting Information

Figure S1 VRP protein expression and serum IgG responses in immunized mice. (A) Western blots showing MNV protein expression from culture supernatants and cell lysates of BHK cells infected with VRPs expressing MNV1.CW3 ORF1, ORF2, or ORF3; Chiba virus (CV) and Lordsdale virus (LV) VLPs were analyzed for cross-reactivity with MNV rabbit polyclonal antisera. VRP-ORF1 infected cells revealed a band corresponding in size to the cleaved RNA-dependent RNA polymerase (57 kDa). VRP-ORF2 infected cell lysate and supernatant both contained capsid protein at high expression levels (58 kDa), and VRP-ORF3 infected cell lysates contained a band corresponding in size to ORF3 (22 kDa) [28],[40]. Purified MNV VLPs from cell lysates yielded a single 57 kDa band corresponding to the capsid. (B) Sera from mice immunized with VRPs expressing ORF2 from MNV1.CW3, Chiba virus (CV), or Lordsdale virus (LV) were tested for cross-reactivity to MNV1.CW3, CV, and LV VLPs by ELISA. (C) Serum anti-MNV antibody by ELISA from adult (8 week old) and aged (14 month old) mice after MNV1.CW3 challenge. These data are pooled from two independent experiments with 3–5 mice per group in each experiment.
(2.77 MB TIF)
