# Supplementary figures and images for: Immune Mechanisms Responsible for Vaccination against and Clearance of Mucosal and Lymphatic Norovirus Infection
Source: PLoS Pathog. 2008 Dec 12;4(12):e1000236. doi: 10.1371/journal.ppat.1000236 (PMC2587711; doi:10.1371/journal.ppat.1000236)

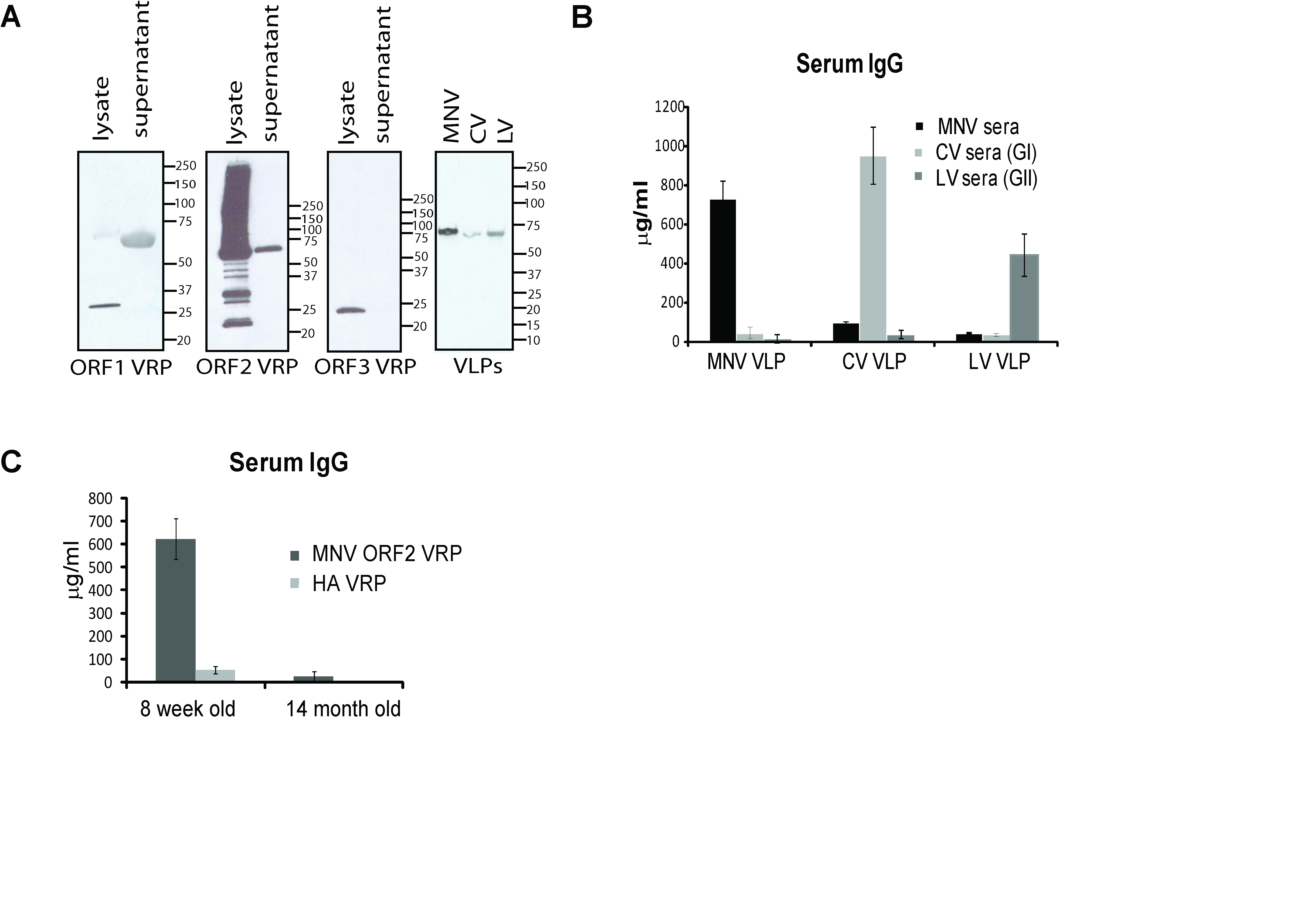

Supplement: Figure S1 — VRP protein expression and serum IgG responses in immunized mice. (A) Western blots showing MNV protein expression from culture supernatants and cell lysates of BHK cells infected with VRPs expressing MNV1.CW3 ORF1, ORF2, or ORF3; Chiba virus (CV) and Lordsdale virus (LV) VLPs were analyzed for cross-reactivity with MNV rabbit polyclonal antisera. VRP-ORF1 infected cells revealed a band corresponding in size to the cleaved RNA-dependent RNA polymerase (57 kDa). VRP-ORF2 infected cell lysate and supernatant both contained capsid protein at high expression levels (58 kDa), and VRP-ORF3 infected cell lysates contained a band corresponding in size to ORF3 (22 kDa) [28],[40]. Purified MNV VLPs from cell lysates yielded a single 57 kDa band corresponding to the capsid. (B) Sera from mice immunized with VRPs expressing ORF2 from MNV1.CW3, Chiba virus (CV), or Lordsdale virus (LV) were tested for cross-reactivity to MNV1.CW3, CV, and LV VLPs by ELISA. (C) Serum anti-MNV antibody by ELISA from adult (8 week old) and aged (14 month old) mice after MNV1.CW3 challenge. These data are pooled from two independent experiments with 3–5 mice per group in each experiment. (2.77 MB TIF) [file ppat.1000236.s001.tif]
